# Supplementary material for: Why people vote for thin-centred ideology parties? A multi-level multi-country test of individual and aggregate level predictors
Source: PLoS One. 2022 Mar 3;17(3):e0264421. doi: 10.1371/journal.pone.0264421 (PMC8893635; doi:10.1371/journal.pone.0264421)
Supplement: S1 File — (DOCX) [file pone.0264421.s001.docx]

**SI Materials -Data**

ESS Round 7 data is available on the Web at <https://www.europeansocialsurvey.org/>).

Populist and Single Issue Parties used in the Dataset

Austria

FPÖ

Team Frank Stronach

Belgium

Lijst Dedecker

Vlaams Belang

Front National

Switzerland

Swiss People's Party

Federal Democratic Union

Czech Republic

KSCM

TOP 09

ANO 2011

ODS

Úsvit prímé demokracie Tomia Okamury

Germany

Die Linke

AfD

NPD

Estonia

Eesti Keskerakond

Eesti Konservatiivne Rahvaerakond

Spain

Unidos Podemos

Podemos

En Comú Podem

Finland

True Finns

Freedom Party

Independence Party

For the Poor

France

FN (Front National)

NPA (Nouveau Parti Anti-Capitaliste)

MPF (Mouvement pour la France)

United Kingdom

UK Independence Party

People Before Profit Alliance

Hungary

Fidesz - KDNP (Fidesz – Magyar Polgári Szövetség Keresztényd)

Jobbik (Jobbik Magyarországért Mozgalom)

Ireland

Anti-Austerity Alliance - People Before Profit

Fianna Fáil

Independents

Israel

HaLikud

Yisrael Beiteinu

Shas

Iceland

Framsóknarflokkinn

Flokk fólksins

Alþýðufylkinguna

Italy

Movimento 5 Stelle

Popolo delle Libertà (PdL)

Lega Nord

Fratelli d'Italia

Lithuania

Lithuanian Freedom Union (Liberals) (LLSL)

Coalition of S. Buškevicius and the Nationalists 'Against corruption and poverty' (Party 'Young Lithuania', Nationalis

Party Order and Justice (TT)

Political Party 'The Way of Courage' (DK)

Lithuanian People's Party (LLP)

Netherlands

Party for Freedom

50PLUS

Norway

Progress Party (FRP)

Poland

KORWIN

Kukiz'15

Law and Justice

Portugal

PNR - Partido Nacional Renovador",

Russian Federation

Fair Russia (SR)

LDPR

Sweden

Sverigedemokraterna

Slovenia

SDS - Slovenska demokratska stranka

**SI Materials-Variables**

In the survey, participants across 23 countries were asked whether they had voted in the last national election. A participant who was eligible to vote and chose [Yes] in response to the question was further asked to select the name of the party he/she had voted for the election (for the names of these parties across the 23 countries in the ESS data the List below). We constructed a dichotomous outcome variable named populist voting (PV: 0 = mainstream party and 1 = populist party) indicating whether an individual voted for a mainstream party or a populist party. In addition to our attitudinal individual-level and country-level variables (i.e., HDI and CPI) we also included demographic variables which we outline below. In the study, an individual-level variable gives information about the participants and may be either a demographic or an attitudinal variable, while a country-level variable provides information about countries such that the same value, i.e., a value of HDI, is assigned to the participants of each country. we elaborate on these (demographic or attitudinal) individual-level variables, and country-level variables that are used to predict the dichotomous outcome populist voting, respectively.

**SI Materials-Testing model assumptions**

Checking model assumptions for a two-level multilevel logistic regression model is more

complicated than that for a single-level logistic regression model, since the former involves checking model assumptions at both levels, that is, the individual-level and country-level for the ESS data. Violating model assumptions at one level may influence the conclusion made when checking model assumptions at another level. We utilize a simulation-based approach to investigate the model assumptions at individual-level using the Dharma package in R. This package provides two main functions: plotQQunif producing a uniform quantile-quantile (QQ) plot on a scale ranging from 0 to 1 and plot. Residuals producing a scatter plot between a set of individual-level residuals and predicted values. We first employ this simulation-based approach when evaluating hypothesis using model $M_{33}$ (see Figure S1). The quantile-quantile plot in this figure (left panel) displays that the distribution of the (scaled) observed residuals ranging from 0 to 1 does not deviate from their expected distribution. The Kolmogorov-Smirnov (KS) test supports this conclusion (i.e., the two sets of residuals come from the same distribution, D = 0.008, *p* = 0.15 > 0.05). On the other hand, the scatter plot (right panel) shows that these uniform residuals between 0 and 1 and the predicted values do not strongly deviate from each other in y-direction (i.e., the red 0.25, 0.50, and 0.75 quantile lines appear to be horizontal). Note that these plots should not be confused with the QQ plots and scatter plots of the level-1 residuals in the context of the standard multilevel regression model containing a continuous outcome, which are created to investigate normality and homoscedasticity (i.e., the constancy of variance) of the residuals, respectively. The plots in this figure do not involve normality or homoscedasticity of the residuals, but instead they show that model $M_{33}$ is correctly specified. That is, the values of the outcome populist voting, the individual-level and country-level predictors (and their cross-level interactions) appear well to be generated from model $M_{33}$ when evaluating hypothesis $H_{3}{}$.

Since the countries are not nested within higher level units, the usual multiple regression diagnostics can be used to evaluate the country-level residuals (i.e., the random intercepts). The multilevel logistic regression assumes that the random intercepts at country-level are normally and independently distributed with a mean of zero. We created three plots to investigate model diagnostics for the random intercepts when evaluating hypothesis $H_{3}$ using model $M_{33}$ (see Figure S2). The quantile-quantile plot (left panel) shows that the assumption regarding the normality of the random intercepts appears to be met, since the values of them fluctuate around the red straight line. The results of the Shapiro-Wilk test support this conclusion that the random intercepts for the 23 countries in the ESS data are normally distributed (W = 0.97, p = 0.76). The random intercept sequence plot (middle panel) is used to visualize a possible autocorrelation (i.e., a serial correlation) between the random intercepts. This plot shows no discernible pattern between the random intercepts as they fluctuate around the zero line randomly. Thus, we conclude that the assumption of independence of the level-2 residuals is reasonably satisfied. In the caterpillar plot (right panel), these residuals are ranked in ascending order and a 95% confidence interval is calculated around each of the residuals. Note that the confidence intervals are quite narrow for almost all the 23 countries in the ESS data, since these countries have large sample sizes (i.e., they contain many individuals). Note furthermore that this plot supports the conclusion that the random intercepts are independent from each other, since most of the confidence intervals do not overlap with each other.

**Appendix**

**F S1**


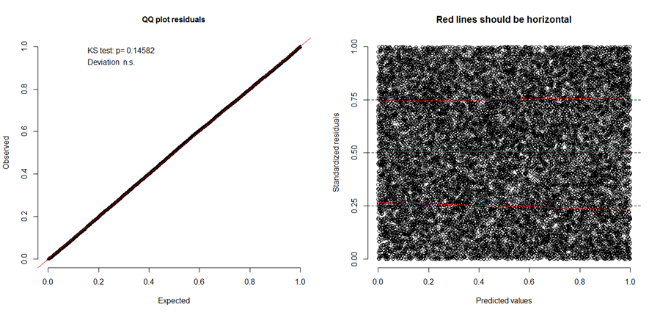


Figure S1. Diagnostic plots for the individual-level residuals when evaluating hypothesis $H_{3}$ using model $M_{33}$.

Figure S2


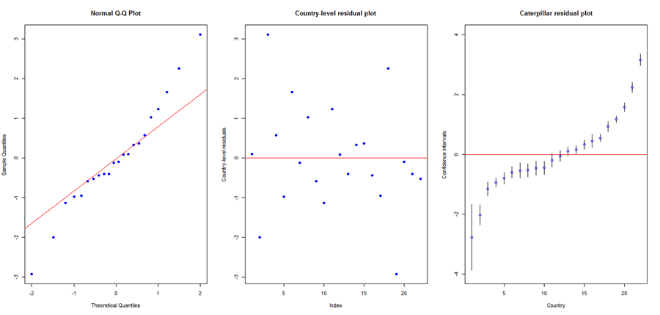


Figure S2. Diagnostic plots for the country-level residuals when evaluating hypothesis $H_{3}$ using model $M_{33}$.
