# Supplementary material for: Why people vote for thin-centred ideology parties? A multi-level multi-country test of individual and aggregate level predictors
Source: PLoS One. 2022 Mar 3;17(3):e0264421. doi: 10.1371/journal.pone.0264421 (PMC8893635; doi:10.1371/journal.pone.0264421)
Supplement: Attachment — Submitted filename: Response to the reviewers R2.docx [file pone.0264421.s003.docx]

Response to the reviewers

Dear Editor,

Thank you very much for the detailed feedback and for the supportive editorial process.

Below, you can find our detailed response to the additional; feedback provided by R2.

Once again, allow us to express our gratitude for the supportive editorial process and the insightful comments which made the manuscript stronger. Please do not hesitate to contact us if you have further comments.

Sincerely,

Huseyin Cakal

R1

R2 comments that *I am happy with how the revised manuscript addresses my comments; the revisions to the framing of the paper allow for more coherence and clarity as to what it is doing, and it helps also in allowing for the discussion to be explicit about its contributions*”

We are most grateful for these encouraging words.

R2 further comments that “*I would only suggest that the authors proofread the changes made. In reading the tracked changes version of the document I came across a number of instances where proof-reading was required. i include only two examples below from the Discussion, but i recommend the authors still read eveyrthing over (or have someone else do so - sometimes we become quite blind to these things when we've been working on a manuscript for a long time!)*

*"by a fundamental intergroup mechanisms, i.e. identity and status concerns which in turn influenced by country level processes" - 'which in turn ARE influenced by'*

*"Results showed that such an effect, if any is contingent to the levels of human development and corruption perceptions at the country level." ''Results showed that such an effect, if any, is contingent to... (usually it's contingent on?')”*

We have proofread the ms and further improved the overall style & edited in these suggestions.
